# The Continuous Wagon Wheel Illusion and the ‘When’ Pathway of the Right Parietal Lobe: A Repetitive Transcranial Magnetic Stimulation Study

**DOI:** 10.1371/journal.pone.0002911

**Published:** 2008-08-06

**Authors:** Rufin VanRullen, Alvaro Pascual-Leone, Lorella Battelli

**Affiliations:** 1 Université de Toulouse, CerCo, UPS, Toulouse, France; 2 CNRS, UMR5549, Faculté de Médecine de Rangueil, Toulouse, France; 3 Berenson-Allen Center for Noninvasive Brain Stimulation, Department of Neurology, Beth Israel Deaconess Medical Center, Harvard Medical School, Boston, Massachusetts, United States of America; 4 Institut Guttmann de Neurorehabilitació, Universitat Autonóma de Barcelona, Badalona, Spain; 5 Vision Sciences Laboratory, Department of Psychology, Harvard University, Cambridge, Massachusetts, United States of America; Indiana University, United States of America

## Abstract

A continuous periodic motion stimulus can sometimes be perceived moving in the wrong direction. These illusory reversals have been taken as evidence that part of the motion perception system samples its inputs as a series of discrete snapshots –although other explanations of the phenomenon have been proposed, that rely on the spurious activation of low-level motion detectors in early visual areas. We have hypothesized that the right inferior parietal lobe (‘when’ pathway) plays a critical role in timing perceptual events relative to one another, and thus we examined the role of the right parietal lobe in the generation of this “continuous Wagon Wheel Illusion” (c-WWI). Consistent with our hypothesis, we found that the illusion was effectively weakened following disruption of right, but not left, parietal regions by low frequency repetitive transcranial magnetic stimulation (1 Hz, 10 min). These results were independent of whether the motion stimulus was shown in the left or the right visual field. Thus, the c-WWI appears to depend on higher-order attentional mechanisms that are supported by the ‘when’ pathway of the right parietal lobe.

## Introduction

Due to the discrete sampling of movie cameras, a wheel on film can sometimes appear to rotate backwards. A similar phenomenon can be perceived in continuous illumination [1], [2], [3], [4], although there are important differences between this “continuous Wagon Wheel Illusion” (c-WWI) and its cinematographic cousin [3], [5], [6], [7], [8]: essentially, the c-WWI is a bistable effect [3], [7] that occurs only sporadically, and requires some adaptation time [5], [9]. Nonetheless, this illusion has been interpreted as evidence that motion perception -or at least one of the numerous motion perception systems [10], [11], [12]- functions by putting together a sequence of discrete snapshots [2], [4], [8], [9], [13], [14], [15], [16]. However, alternative explanations have been proposed, suggesting that the illusion relies on the spurious activation of low-level motion detectors which, after sufficient adaptation time, might come to dominate perception [3], [6], [7]. One of the major differences between these two accounts of the illusion is the level at which it would be triggered: higher-level motion processing areas for the “snapshot” hypothesis, vs. lower-level areas for the alternative. Of course, it is also possible that both low-level and high-level factors could jointly contribute to the phenomenon. Our previous work has shown that the c-WWI effect depends on attentional [4], object-based [14] mechanisms, and that it occurs similarly for first-order (luminance-defined) and second-order (contrast-defined) motion [4]: this already casts doubt on a simple low-level explanation. Here we further test the hypothesis of a high-level contribution to the c-WWI.

Further electro-encephalographic (EEG) investigation has revealed a single correlate of the illusion, in a frequency band compatible with the predictions of the “snapshot” hypothesis, and specifically localized over right parietal electrodes [13]. This result provides us with a prime candidate region to explore as the source of the c-WWI effect.

The right parietal lobe is involved in attending to visual events that are displaced in space and time. In particular, patients with lesions of the right inferior parietal cortex, and suffering from left visual neglect, have difficulties in perceiving long-range apparent motion [17], or in judging the temporal direction (onset vs. offset) of luminance transients [18]. Interestingly, these deficits are observed for stimuli placed in either visual field (and not just on the left, the field contralateral to the lesion), and do not occur following lesions of the left parietal lobe. These and other results have led to the proposal that the right inferior parietal lobe (IPL) supports our temporal perception of the world, acting as part of a *when* pathway [19]. In this view, parietal areas are generally involved in attentional processes as part of the “where” pathway, but the right parietal lobe additionally takes on the task of attending to temporal aspects of the world, i.e. “when” information. Thus, if the c-WWI is due, at least in part, to discrete temporal sampling of motion information, a mechanism potentially involved in apparent motion perception, we hypothesized that this sequencing would depend on a function of the right IPL. Alternatively, if the illusion simply reflects the spurious activation of low-level motion detectors, there would be no reason to predict a specific involvement of right vs. left parietal regions. By comparing the effect of low frequency repetitive transcranial magnetic stimulation (rTMS) applied over the left and right IPL on the intensity of the c-WWI, we will thus be able to reveal a possible (though not necessarily exclusive) involvement of higher-level processes. This would not preclude the “intermediate” possibility of both low-level and higher-level factors jointly contributing to this illusory phenomenon, but it would allow us to rule out the simplistic low-level account.

## Results

**Figure 1:**
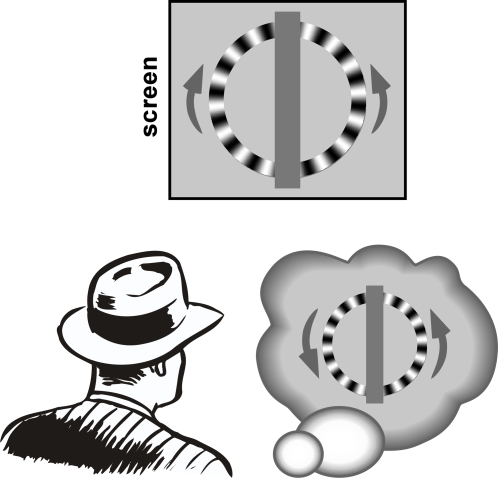
The c-WWI effect. When viewing periodic motion around 10 Hz in continuous illumination (or using a monitor with a fast enough refresh rate), an illusion of reversed motion can sometimes occur. Here we used an annulus stimulus made of two halves rotating in opposite directions, which can facilitate the occurrence of perceptual reversals in one half (making the entire pattern appear to rotate clockwise) or in the other (making it appear to rotate counter-clockwise, as represented here). The subject's task was to report motion reversals by pressing the corresponding key (right finger for the right half of the stimulus and left finger for the left half) for as long as the reversal lasted. A movie rendition of this stimulus can be viewed at: http://www.cerco.ups-tlse.fr/rufin/ringmovies/.

The stimulus was an annulus split vertically in the middle, each half containing a radial luminance grating rotating at 10 Hz –the optimal frequency for obtaining illusory reversals [4]. The left and right halves of the annulus rotated in opposite directions (one clockwise, the other counterclockwise, counterbalanced across subjects), creating an inconsistent global motion pattern that was resolved when either the left or the right half reversed (Figure 1). We showed previously that this is an effective way of maximizing the occurrence of the c-WWI [14]. In addition, because this type of stimulus can reverse separately in the left or the right hemifield, it was an ideal choice for us to study the potential lateralization of the changes induced by unilateral rTMS.

Subjects (n = 6) saw the stimulus for one minute, during which they reported when the left or the right side of the pattern (or both) appeared to reverse, by holding down one of two pre-assigned keys (or both). The strength of the c-WWI was measured as the percentage of viewing time spent with an illusory percept, i.e. with at least one key held down. Subjects performed 5 one-minute trials in a row, each separated by a rest period of one minute. These 9-minute sessions were collected under 4 different experimental treatments. All subjects started with a baseline session, which was followed by 10 minutes of 1 Hz rTMS on the left or the right IPL, with the order of stimulation counterbalanced across subjects. Immediately after the stimulation, a new (2^nd^) experimental session was collected. After 15 minutes rest, the homologous brain area on the opposite side of the head was stimulated for 10 minutes, followed immediately by another (3^rd^) experimental session. Finally, after a final 15 minutes break, we collected a last baseline session (4^th^), which established whether performance had returned to pre-TMS levels.

**Figure 2:**
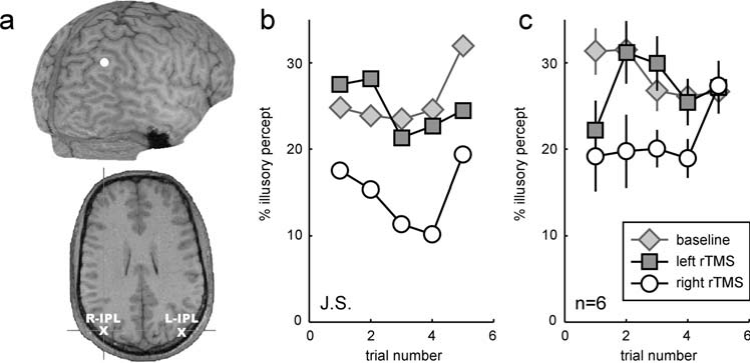
Effect of rTMS on the c-WWI. a. Targeted anatomical locations for one representative naive subject. Top: white dot indicates the right hemisphere stimulation site on the 3-D reconstruction. Bottom: transverse section showing the projected anatomical locations for the right (R-IPL) and left (L-IPL) inferior parietal lobules respectively. b. Experimental results of the same subject. Each trial lasted one minute and was followed by one minute of rest. Baseline performance was collected both at the beginning and at the end of the experimental session (for clarity the two curves have been collapsed here). The percentage of time that the subject spent reporting an illusory (reversed) percept was diminished after rTMS of the right IPL, but not following stimulation of the left IPL. c. Average results of 6 subjects. Error bars report the s.e.m. There was a significant main effect of stimulation condition (p<0.001) which was due to the right rTMS stimulation significantly lowering the strength of the c-WWI relative to left rTMS or the baselines (tukey-kramer multiple comparisons post-hoc test). There was no main effect of trial number, and no significant interaction between the 2 factors.

The strength of the illusion (proportion of viewing time spent with an illusory percept) was evaluated as a function of time (trial number 1–5) and experimental treatment (first baseline, last baseline, left and right rTMS) using a 2-way ANOVA (Figure 2). There was a significant main effect of experimental treatment (F(3,100) = 6.0, p<0.001), due to a significantly weaker illusion following right rTMS than in any of the other conditions, which were not significantly different from one another (post-hoc Tukey-Kramer multiple comparisons test, alpha = 0.05). Note that the absence of a difference between the first and last baseline periods indicates that the effects of rTMS had receded by the end of the experiment. There was no significant main effect of trial number (F(4,100) = 0.9, p = 0.47), nor any interaction between trial number and experimental condition (F(12,100) = 1.0, p = 0.45). This lack of significant interaction is likely to reflect insufficient statistical power, since it is apparent upon observing Fig 2c that any behavioral effect of rTMS has returned to the baseline level by the 5^th^ trial (i.e. ∼8 minutes after the end of the stimulation period). Similarly, the presence of an illusory percept decrement during the first trial following left rTMS is suggested by the data in Figure 2c, even though it does not appear as a significant trial × condition interaction in the ANOVA. Such an effect could be explained by a transient global disruption of performance following rTMS, and is much smaller and short-lived in comparison with the major effect observed following right rTMS. To summarize, repetitive stimulation of the right IPL, but not of the left, significantly decreased the c-WWI (Figure 2). Since the parameters we used for rTMS are known to induce a transient deactivation of the stimulated area [20], this supports the idea that right inferior parietal regions normally contribute to the c-WWI.

**Figure 3:**
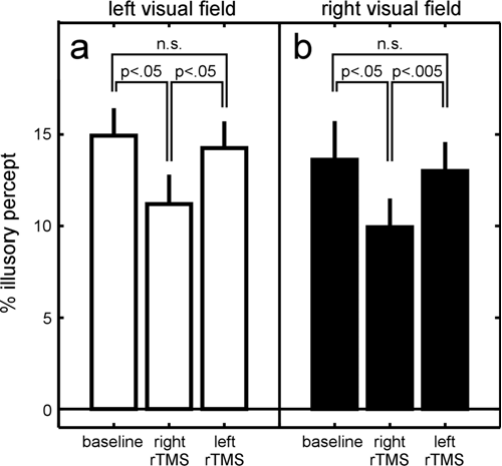
Bilateral effect of rTMS over right IPL. a. Illusory reversals (proportion of total viewing time) occurring in the left hemifield in the three different experimental conditions (average of 6 subjects). b. Illusory reversals occurring in the right hemifield. There was no significant difference between the two hemifields. In both cases, stimulation of right IPL induced an illusion that was significantly weaker than baseline or left rTMS, which were not significantly different from each other.

We then asked whether the effect obtained following right IPL stimulation was bilateral, or limited to the contralateral visual field. To this end, we separately considered the illusory reversals that occurred in the left and in the right visual field (Figure 3). Repeating our previous ANOVA with the additional factor “left/right visual field”, we found again a main effect of experimental condition (F(3,200) = 6.3, p<0.0005), but no main effect of trial number (F(4,200) = 1.0, p = 0.4) or left/right visual field (F(1,200) = 3.1, p>0.05), and no significant 2-way or 3-way interaction (all p>0.05). Post-hoc paired t-tests revealed that in the left visual field, right rTMS significantly decreased the strength of the illusion compared to baseline (collapsed over the two baseline periods; t(5) = 2.83, p<.05) or compared to left parietal rTMS (t(5) = 3.31, p<.05). The same results were also found in the right visual field (right rTMS vs. baseline, t(5) = 3.19, p<.05; right rTMS vs. left rTMS, t(5) = 6.56,p<.005). In both visual fields, the illusion following left rTMS was not significantly different from baseline (t(5)<1.0, p>.05). To conclude, the effects of rTMS on a given side of the brain (when present) are observed bilaterally, and not just contralaterally, therefore the effect is not spatially lateralized after unilateral stimulation.

## Discussion

The present study is the first to date to directly link a particular brain region to the continuous Wagon Wheel Illusion (c-WWI). The c-WWI was significantly and bilaterally weakened following slow frequency rTMS, and hence deactivation, of right IPL, but no effect was found after stimulation of the homologous left region. Illusory reversals, however, were not completely annihilated by rTMS. This might reflect an incomplete deactivation of the cortical areas under study. Indeed studies in humans and in animal models suggest that as applied rTMS will lead to a suppression of activity in the targeted brain region by approximately 30% [21], [22]. Alternatively, the incomplete disruption of the illusory reversal might indicate that other, potentially lower-level areas also participate in this illusion, as suggested in alternative accounts of the illusion [3], [6], [7]. At present there is insufficient data to decide between these two alternatives. Nevertheless, the findings as a whole support and extend our previous observations of EEG correlates of the c-WWI over right parietal electrodes [13], by demonstrating, for the first time, a clear causal role for right parietal cortex in the generation of the c-WWI effect. Spurious activation of low-level motion detectors, as proposed by other authors [3], [6], [7] could not, by itself, account for this pattern of results.

An alternative explanation of our result is that TMS might have simply disrupted a mechanism that is responsible for bistable switches (independent of where and how the percepts are generated) [23], [24]. Compatible with this idea, a recent study using binocular rivalry on left visual neglect patients, generally affected by a right IPL lesion [27] has shown that these patients had a much slower perceptual alternation of two rivalrous gratings presented foveally compared to healthy controls and patients without neglect [26]. However, a recent rTMS study using a similar TMS procedure as in our study showed that when TMS is delivered over the right posterior parietal cortex, the rate of switching of two rivalrous stimuli is actually *increased* immediately after stimulation (TMS over the left homologous area had no effect) [25]. This is the exact opposite effect to what we found in our study, where the rate of perceptual switching was significantly decreased after TMS over the right parietal cortex (expressed as a significant reduction of the illusion). Thus, in the absence of a clear unequivocal link between inactivation of right IPL and alterations of bistable switching rate, we believe that the impairment we found in the present study was likely related to an event timing mechanism temporarily disrupted by TMS [19]. Moreover, the detrimental effect of right IPL rTMS on the illusion was observed bilaterally. This points to a rather high-level process, and adds to the existing list of non-spatial temporal functions of the so-called ‘when’ pathway of the right parietal lobe [19].

Essentially, if the ‘snapshot’ hypothesis is correct, then one implication of our results could be that right parietal regions serve to decompose the incoming temporal stream into a sequence of discrete events, upon which our temporal perception of the world would be constructed. Hence, temporally separated flashes at distant locations could be bound into an apparent motion percept [17], [28]; transient visual events in rapid succession could be categorized as simultaneous or sequential [18], [29]; but when the rate of presentation of a periodic display falls within the “wrong” range, this temporal sampling would inopportunely induce an erroneous percept: the continuous Wagon Wheel Illusion. A future step in demonstrating this assumption could involve “online” disruption of the illusion by precisely-timed single-pulse TMS, thus capitalizing on the high temporal resolution of the technique.

## Methods

### Participants

Two authors (RV and LB) and four naïve subjects took part in the study, which was conducted according to the ethical guidelines of the Internal Review Board at the Beth Israel Hospital. All had normal or corrected-to-normal vision. Before the start of the experiment, all naïve subjects were familiarized with the illusion, and received a few practice trials with the stimulus and task (between 2 and 5 minutes depending on the subjects). Two additional subjects were tested but had their data rejected on the basis of their baseline performance. Their baseline percentage of illusory duration was higher than the group mean (i.e. 28.5%±6.5%) by more than 3 standard deviations for one subject and more than 5 standard deviations for the other, and therefore they were regarded as outliers and their data were excluded from the analysis. All subjects signed a written informed consent form.

### Visual stimulation

The stimulus was a radial grating (24 cycles) at maximum contrast, displayed in an annulus of width 1 degree starting at 4 degrees eccentricity (Fig 1). The vertical midline of the annulus was occluded by a vertical grey band, on which a fixation point was shown throughout the experiment. Subjects were instructed to fixate and to refrain from making eye movements. For a given subject, one half of the annulus always rotated clockwise, the other counterclockwise; this was counterbalanced across subjects. The temporal frequency of the rotation was 10 Hz in both halves of the stimulus. A given trial lasted 60 s with constant visual stimulation, during which subjects were required to hold down the left SHIFT key on the keyboard whenever the left half of the stimulus appeared to reverse, and to hold down the right SHIFT key if an illusory reversal occurred in the right half. If both halves reversed together, both keys could be pressed simultaneously. However, the design of our stimulus implied that this was extremely rare (less than 0.05% of total viewing time on average).

### Experimental protocol

An experimental session consisted of 5 trials as described above (60 s each), separated by rest periods of 60 s, for a total of 9 minutes. Each subject performed 4 such experimental sessions. Subject's baseline performance was always measured during the first session. One side of the brain (randomly assigned for each subject: left parietal cortex for 3 of them, right parietal for the other 3) was then stimulated using 1 Hz rTMS for 10 minutes, after which a second experimental session was collected. Stimulation was then performed on the other side (1 Hz, 10 min), followed by a third experimental session. Finally, after a 15 min rest period, subjects performed a fourth experimental session, which served as a baseline and allowed us to verify that any rTMS-induced effects had receded.

Note that, for convenience, eye movements were not recorded during this experiment. However, previous studies have shown that the illusion does not depend on the occurrence of eye movements (e.g. Purves, et al. 1996).

### TMS Protocol

TMS was delivered using a MagStim stimulator (MagStim, Whitland, UK) and a 70 mm figure-of-eight Magstim stimulation coil. We applied a 10 min train of repetitive low-frequency (1 Hz) stimulation over one of the two brain sites, right IPL or left IPL. The intensity of stimulation was set at 75% maximum stimulator output like in previous successful studies [30]. Each subject underwent one testing session and the order of stimulation was counterbalanced across subjects. Previous studies have shown that 1 Hz stimulation temporarily reduces excitability of the cortex (within the stimulated area) and the excitability effects outlast the period of stimulation [31]. The coil was held with the handle pointing backward toward the back of the head and positioned perpendicular to the stimulated region.

Immediately following the repetitive stimulation over the targeted brain site, subjects performed the task (same task as the Pre-Stimulation Baseline). The time required to perform the task (approximately 10 min) is within that for which rTMS has been shown to have lasting effects in parietal regions [32]. After completion of the task, observers rested for 15 min to allow complete recovery from the stimulation. Stimulation was then applied to the remaining brain site in the opposite hemisphere, and the subject again performed the task.

### Brain Localization

High resolution anatomical images in conjunction with frameless stereotaxy (BrainSight™, Rogue Research, Montreal, Canada) were used to visualize the projected cortical target of the right and left IPL stimulation sites in three subjects (Figure 2a, example of subject JS). The projected target of stimulation over IPL corresponded to the angular gyrus, the same regions also implicated in visual timing and high-level apparent motion deficits in parietal patients [17]. For the remaining three subjects to aid in brain site localization, subjects wore a lycra swimmer's cap with a reference point positioned over the inion. Right and left IPL were localized as P4 and P3 respectively using the EEG 10/20 system. However, since we wanted to deliver rTMS over the IPL and not on the superior parietal lobe (P4 and P3 are located on the posterior portion of the superior parietal lobe) we successively moved 1.5 cm posterior and 2 cm across from P4 to localize the IPL. This was done after we performed the same 10/20 coordinate measurements for those three subjects we had the anatomical MRI and we ascertained the exact locations with the frameless sterotaxy system.
